# Randomized trial of same- versus opposite-arm coadministration of inactivated influenza and SARS-CoV-2 mRNA vaccines

**DOI:** 10.1172/jci.insight.187075

**Published:** 2025-01-09

**Authors:** Wen Shi Lee, Kevin J. Selva, Jennifer Audsley, Helen E. Kent, Arnold Reynaldi, Timothy E. Schlub, Deborah Cromer, David S. Khoury, Heidi Peck, Malet Aban, Mai Ngoc Vu, Ming Z.M. Zheng, Amy W. Chung, Marios Koutsakos, Hyon-Xhi Tan, Adam K. Wheatley, Jennifer A. Juno, Steven Rockman, Miles P. Davenport, Ian Barr, Stephen J. Kent

**Affiliations:** 1 Department of Microbiology and Immunology and; 2 Department of Infectious Diseases, Peter Doherty Institute for Infection and Immunity, University of Melbourne and Royal Melbourne Hospital, Melbourne, Victoria, Australia.; 3 Kirby Institute, University of New South Wales, Kensington, New South Wales, Australia.; 4 Sydney School of Public Health, Faculty of Medicine and Health, University of Sydney, Sydney, New South Wales, Australia.; 5 WHO Collaborating Centre for Reference and Research on Influenza, Royal Melbourne Hospital, Peter Doherty Institute for Infection and Immunity, Melbourne, Victoria, Australia.; 6 Vaccine Innovation Unit, CSL Seqirus Ltd, Parkville, Victoria, Australia.; 7 Melbourne Sexual Health Centre and Department of Infectious Diseases, Alfred Hospital and Central Clinical School, Monash University, Melbourne, Victoria, Australia.

**Keywords:** Clinical trials, Vaccines, Influenza

## Abstract

**BACKGROUND:**

The immunogenicity of current influenza vaccines needs improvement. Inactivated influenza and COVID-19 mRNA vaccines can be coadministered, but randomized controlled trial data are lacking on whether the 2 vaccines are more immunogenic if given in the same arm or opposite arms. Murine studies suggest mRNA vaccines can adjuvant influenza vaccines when coformulated and codelivered.

**METHODS:**

We randomly assigned 56 adults to receive the Afluria quadrivalent inactivated influenza and Moderna monovalent SARS-CoV-2 XBB.1.5 mRNA vaccines, either in opposite arms or both in the same arm at the same site. The primary endpoint was the difference in median combined serum hemagglutination inhibition titer to the H1, H3, and B-Vic vaccine influenza strains after vaccination.

**RESULTS:**

We found no significant difference in hemagglutination inhibition antibody levels between the groups (*P* = 0.30), with the same-arm group having a 1.26-fold higher titer than the opposite-arm group. There were no differences in analyses of antibodies against individual influenza strains or in nasal or saliva antibody levels. While both binding and neutralizing antibody titers against SARS-CoV-2 were not significantly different between groups postvaccination, there was a higher fold-change in BA.5 and ancestral strain neutralizing antibodies in the opposite-arm group.

**CONCLUSION:**

Influenza vaccination is equivalently immunogenic if given in the same arm or opposite arms as the SARS-CoV-2 vaccine, but it may be preferable to administer the SARS-CoV-2 vaccine at a different site from influenza vaccines.

**TRIAL REGISTRATION:**

Australian New Zealand Clinical Trials Registry ACTRN12624000445572.

**FUNDING:**

Australian National Health and Medical Research Council, Australian Medical Research Future Fund, and National Institutes of Health (UH2AI176172).

## Abstract 2

Should you get the COVID and Influenza vaccines in the same arm or opposite arms? Antibody responses to influenza were similar with either strategy.

## Graphical abstract

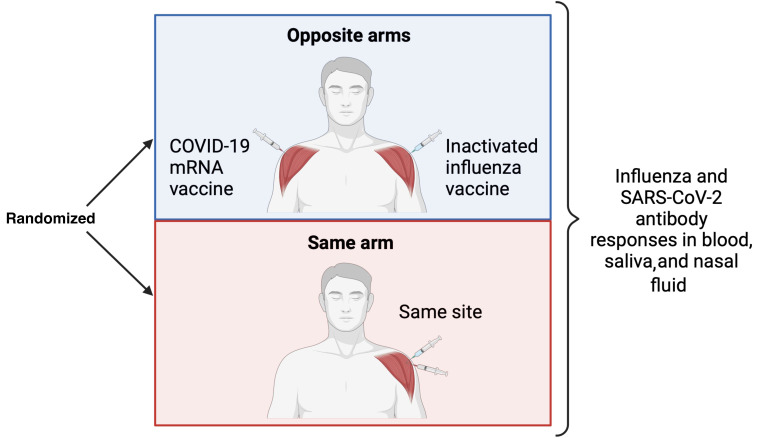

## Introduction

There is a need to safely improve the immunogenicity and efficacy of the standard unadjuvanted inactivated influenza vaccine (1, 2). Use of the MF59 adjuvant or a higher dose improves efficacy, but the effect is modest and generally recommended only for older or immunocompromised participants since such vaccines are more expensive and reactogenic (3, 4). mRNA lipid nanoparticle vaccines for SARS-CoV-2 induce high levels of protective immune responses (5). The ionizable lipid component of the lipid nanoparticle is a potent adjuvant of mRNA vaccines (6). Indeed, in the context of primary immunization, ionizable lipids have a robust adjuvant effect on protein vaccines in animal studies (7).

The antigenic drift of both influenza and SARS-CoV-2 viruses has led to regular reformulation of both vaccines to incorporate more recently circulating strains. Influenza vaccines are recommended annually, and regular SARS-CoV-2 boosters are also commonly recommended (8). Since both unadjuvanted influenza vaccines and SARS-CoV-2 mRNA lipid nanoparticle boosters can be given annually, it may be practical and lead to higher community uptake if both vaccinations are given at the same medical visit. The question then arises if the 2 vaccines should be given in the same arm or opposite arms. Most jurisdictions advise that either the same arm or opposite arms can be used, since there are little data available to recommend one strategy over the other (9).

Two recent observational studies suggested minimal differences in either influenza- or SARS-CoV-2–specific antibody levels elicited with same- or opposite-arm immunization (10, 11). However, those studies were not randomized and the selection of participants could introduce potential confounders. In one study, most people preferred opposite-arm vaccination, and the time intervals of sampling were wide (10). Furthermore, the precise location of both vaccines given in the same-arm immunization was unclear. As noted above, murine studies show an ionizable lipid can adjuvant an influenza protein vaccine if given in the same location (7). This raises the hypothesis that the ionizable lipid within the SARS-CoV-2 mRNA lipid nanoparticle vaccine could act as an effective adjuvant to the influenza vaccine if given at the same location in the same arm. If effective, a same-site vaccination strategy could enhance the effectiveness of the unadjuvanted influenza vaccine without the need for a more costly adjuvanted or high-dose vaccine.

We conducted a randomized controlled trial of administering the unadjuvanted 2024 quadrivalent inactivated influenza vaccine (Afluria) on the same day as the XBB.1.5 monovalent SARS-CoV-2 mRNA booster (Spikevax) either at the same site in the same arm or in opposite arms. The primary outcome was the serum hemagglutination inhibition (HI) titers to influenza strains within the vaccine.

## Results

**Figure 1:**
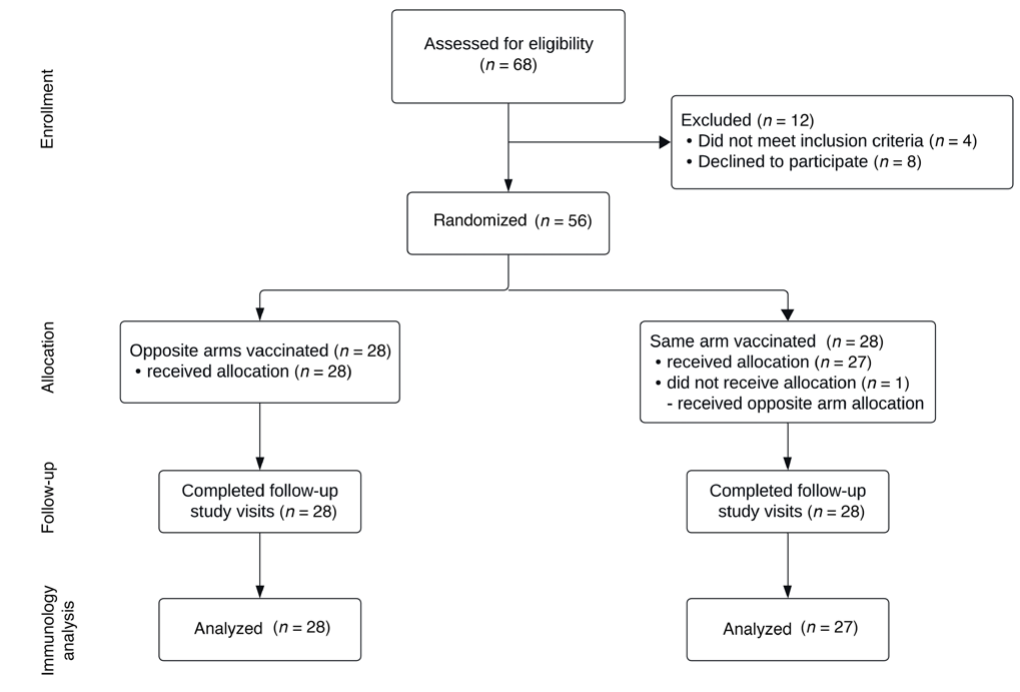
CONSORT flow diagram describing trial recruitment for the CANNON study. CANNON, Covid-19 ANd iNfluenza vaccinatiON.

*Clinical trial*. Fifty-six adults were recruited between March 8 and April 9, 2024, and followed for 28 days. One participant randomized to the same-arm allocation received the vaccines in opposite arms and was therefore excluded from analysis (Figure 1). Participants were stratified for age, sex, and receipt of 2023 influenza immunization. Demographics showed no major differences between the 2 treatment groups (Table 1). All individuals completed the study. The vaccines had an expected safety profile, with the same-arm immunization group having 67 total adverse events reported, compared with 52 for the opposite-arm group (Table 2). The total cohort reported 119 adverse events, with 50/55 (91%) of participants reporting at least 1 local reaction and 29/55 (52.7%) reporting at least 1 systemic reaction. All adverse events were grade 1 or 2, except for a single report of pain at injection site that was grade 3 (prevented daily routine). That individual was randomized to receive the vaccine in opposite arms, and the grade 3 reaction occurred in the nondominant (SARS-CoV-2 vaccine) arm. There was no overall significant difference in the proportion of participants reporting local or systemic reactions between the same-arm and opposite-arm vaccine groups (*P* = 0.35 and *P* = 0.79, respectively, Fisher’s exact test).

Although local pain was reported by almost all participants, the same-arm group had a larger number of local swelling and redness events reported compared with the opposite-arm group (a total of 9 events reported by 6 participants vs. 2 events reported by 1 participant, respectively). This was likely related to both vaccines being given at the same site. In the opposite-arm vaccination group, the proportion of individuals reporting at least 1 local reaction (pain, redness, and/or swelling) was significantly higher (*P* = 0.006, Fisher’s exact test) in the nondominant arm (SARS-CoV-2 vaccine) compared with the dominant arm (influenza vaccine), as expected since local reactions are more common with the SARS-CoV-2 mRNA vaccine (Table 2).

**Figure 2:**
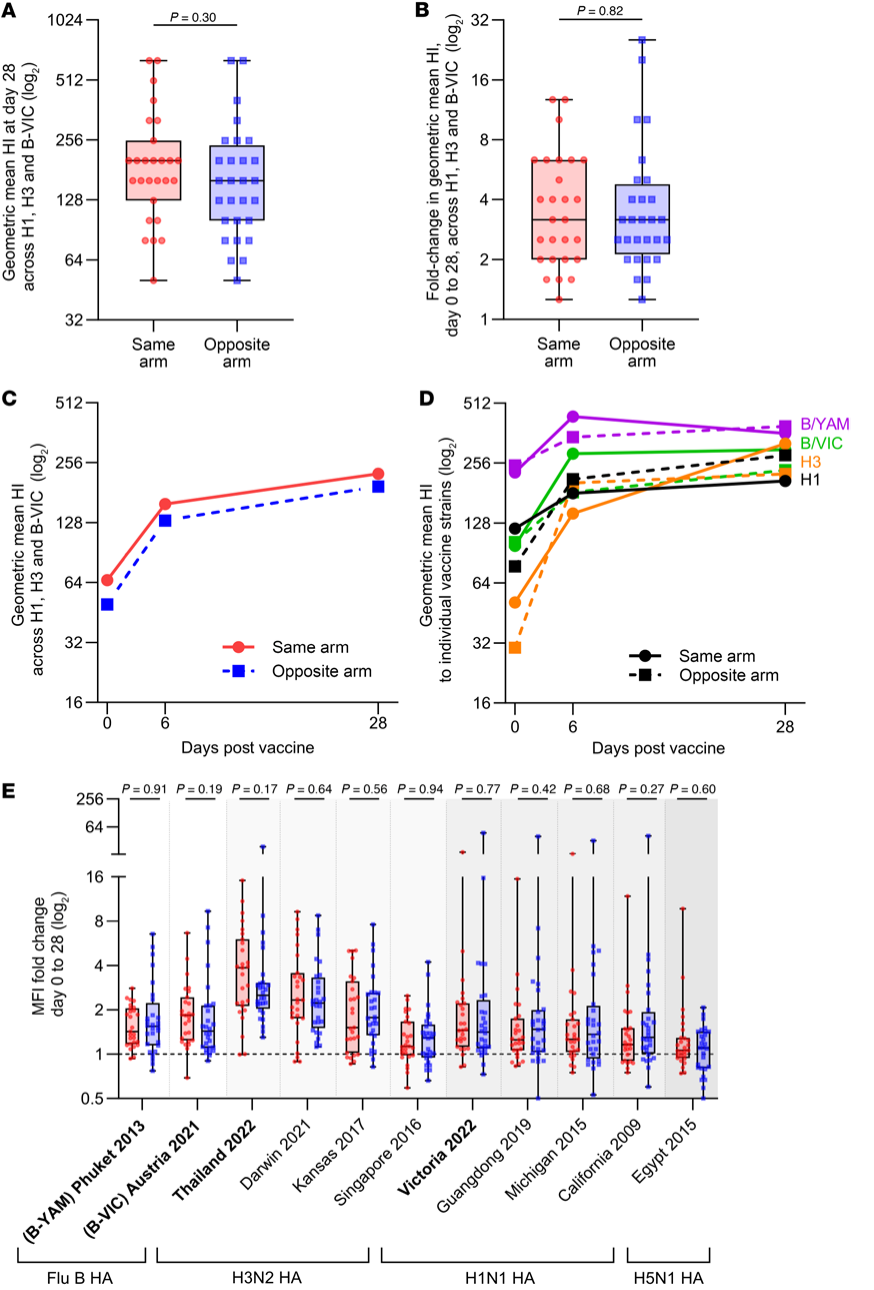
Plasma antibody responses to influenza following coadministration of COVID-19 mRNA boosters and inactivated influenza vaccines in the same or opposite arms. (**A** and **B**) Geometric means of HI titers across 3 strains (H1N1, H3N2, and B-VIC) included in the influenza vaccine for both the same-arm (*n* = 27; red circles) and opposite-arm (*n* = 28; blue squares) groups. Box-and-whisker plots compare either (**A**) responses at day 28 alone or (**B**) the fold-change in geometric means of HI titers from day 0 to day 28 postvaccination. Statistical significance was calculated between groups using the 2-tailed Mann-Whitney *U* test. (**C**) Line graphs depict the geometric mean HI titers for each group at days 0, 6, and 28 postvaccination (averaged across individuals and the 3 vaccine strains H1N1, H3N2, and B-VIC). (**D**) Line graphs show the geometric mean HI titers for each vaccine strain (H1N1, H3N2, B-VIC, B-YAM), averaged across individuals. The same-arm cohort is depicted in solid lines, while the opposite-arm cohort is in dotted lines. (**E**) Fold-change in plasma IgG antibody binding levels against recombinant HA proteins from different circulating influenza strains as measured by bead-based multiplex (final dilution 1:25,600). Vaccine strains are indicated in bold. Statistical significance was calculated between groups using the 2-tailed Mann-Whitney *U* test. For box-and-whisker plots, central lines indicate medians, boxes indicate 25th and 75th percentiles, and whiskers indicate range (minimum and maximum).

*Influenza antibody levels*. Our hypothesis was that immunizing at the same site could boost influenza immunity through provision of an adjuvant by the lipid nanoparticle vaccine at the same site. The primary endpoint was the fold-change in combined geometric mean of serum HI titers to the 3 circulating influenza strains within the vaccine (H1, H3, and B-Vic strains) 28 days after vaccination, in the same-arm group compared with the opposite-arm group. We found nearly identical levels of HI antibodies in the same-arm and opposite-arm groups, with no significant differences (1.26-fold higher [95% CI = 0.78–1.43] in same-arm group; *P* = 0.30; Figure 2A). There was a 3.2-fold increase in HI antibodies against these 3 strains in both the same- and opposite-arm groups, with no significant difference between the groups (*P* = 0.82; Figure 2B). Analysis of HI levels over time (Figure 2C) and to individual influenza vaccine strains (Figure 2D), including the B-Yam strain in the vaccine, also showed no differences between the groups.

Although the influenza vaccine strain antibody immunity was not different between the arms, it was possible that immunity may have been broader in one or the other arms. We therefore analyzed whether immunity to nonvaccine strains was preferentially boosted in either the same- or opposite-arm group by using a bead-based multiplex array to compare total binding antibody levels (Supplemental Figure 1; supplemental material available online with this article; https://doi.org/10.1172/jci.insight.187075DS1). This analysis verified the lack of difference in antibody responses to the vaccine strain HAs and showed no difference in antibody levels against 6 additional nonvaccine H1 and H3 proteins circulating in previous years (Figure 2E and Supplemental Figure 2, A, C, and E). Prior to vaccination, there was a general hierarchy of responses toward older strains in both arms (Supplemental Figure 2, A, C, and E). Unsurprisingly, the vaccine induced minimal responses to H5 proteins, with no differences between the arms to the recently circulating 2.3.4.4b clades (Texas 2024; S. Carolina 2021) (Figure 2E and Supplemental Figure 3, A, D, and G).

**Figure 3:**
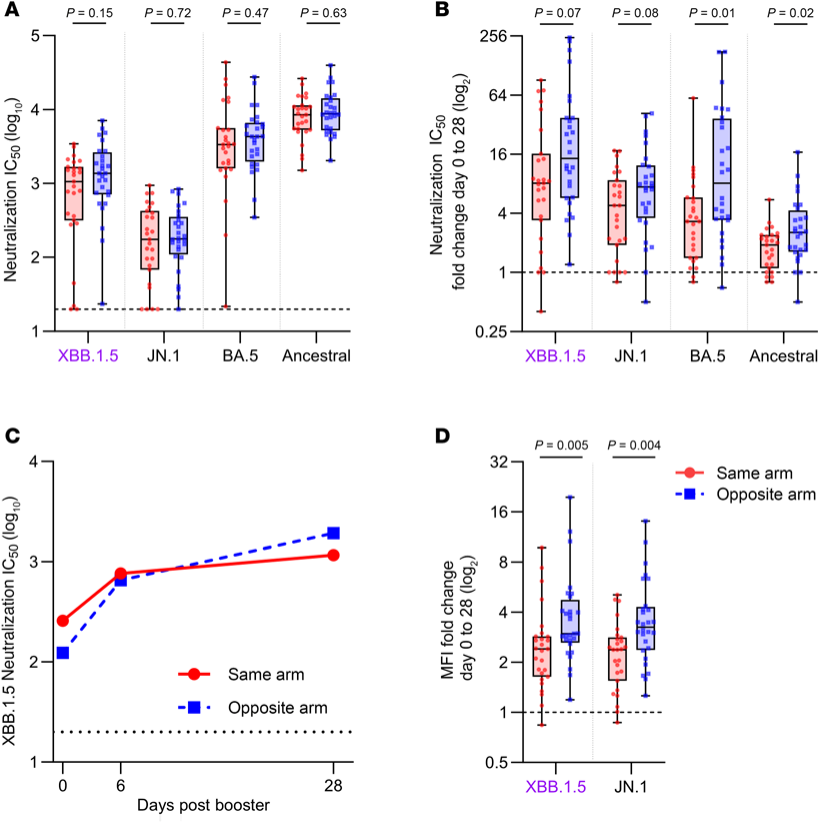
Neutralizing antibody responses to SARS-CoV-2 in plasma between the same- or opposite-arm groups. (**A** and **B**) Box-and-whisker plots show plasma neutralization activity (IC_50_) in a live virus neutralization assay against the vaccine strain XBB.1.5 (in purple) and other SARS-CoV-2 strains (JN.1, BA.5, and ancestral) (**A**) at 28 days alone or (**B**) as a fold-change of responses between day 0 to day 28 postvaccination. (**C**) Line graphs illustrate the geometric mean plasma neutralizing titers against XBB.1.5 for each vaccine group at days 0, 6, and 28 postvaccination (accounting for values below the detection limit). (**D**) Box-and-whisker plots depict the fold-change in plasma IgG antibody binding levels to XBB.1.5 and JN.1 spike proteins as measured using a bead-based multiplex assay (final dilution 1:25,600). The same-arm group (*n* = 27) is represented by red circles while the opposite-arm group (*n* = 28) is shown as blue squares. Box-and-whisker plots show the interquartile range (box), median (line), and minimum and maximum (whiskers). Experiments were performed in duplicate. Statistical significance was calculated between groups using the 2-tailed Mann-Whitney *U* test.

*SARS-CoV-2 antibody levels*. Our primary hypothesis was that the COVID-19 mRNA vaccine could boost the influenza vaccine response, but influenza vaccination could instead affect the response against SARS-CoV-2. We used a live virus assay to evaluate plasma neutralizing antibody responses to the vaccine strain (XBB.1.5), a more recent circulating strain (JN.1), and older strains included in previous vaccines (ancestral and BA.5) (Figure 3A). Our prespecified secondary outcome was the fold-rise from day 0 to day 28 of the neutralizing antibody titer to XBB.1.5 and other strains comparing between the same-arm and opposite-arm groups. We found a significantly higher fold-rise in antibody titers in the opposite-arm group only for BA.5 and ancestral strain (*P* = 0.01 and 0.02 respectively; Figure 3B). However, when looking at the absolute neutralization titers, which are the predictors of protection, there were no significant differences in the day 28 neutralizing antibody titers to XBB.1.5 and other variants (Figure 3B). The opposite-arm group started with slightly lower day 0 neutralizing antibody titers against BA.5, contributing to the higher fold-rise following vaccination, though the baseline titers were not significantly different between groups for any of the strains tested (Supplemental Figure 4, A–D).

A time course of the rise of XBB.1.5 neutralizing antibody response is shown in Figure 3C, with the rise in XBB.1.5 neutralizing antibodies being detectable by day 6, indicating rapid recall of preexisting memory responses. We verified the neutralizing antibody results using a bead-based multiplex ELISA and found higher fold-increase in total plasma IgG antibody responses from day 0 to day 28 in the opposite-arm group to both the XBB.1.5 and JN.1 spike proteins (Figure 3D). However, like the absolute neutralizing antibody titers, total IgG titers against XBB.1.5 and JN.1 spike proteins were not significantly different at baseline or at day 28 postvaccination (Supplemental Figure 2, B, D, and F).

*Mucosal antibody levels*. Mucosal immunity forms a protective barrier against infection by respiratory viruses, such as influenza and SARS-CoV-2. We have previously shown that while intramuscular vaccinations were poor at inducing local mucosal IgA antibodies, they could elevate IgG antibodies in mucosal secretions, likely via transudation from blood (12). Here, we investigated if mucosal IgG antibodies against influenza and SARS-CoV-2 in saliva and nasal fluid would differ following administration to different immunization sites.

**Figure 4:**
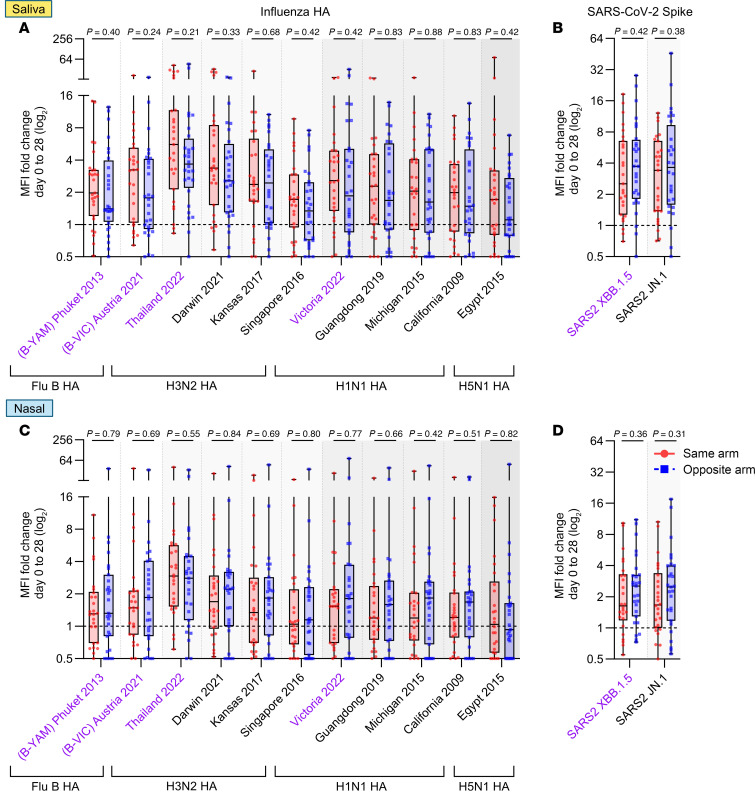
Salivary and nasal IgG antibody binding responses to influenza HA and SARS-CoV-2 spike following vaccination. Box-and-whisker plots illustrate the fold-change in IgG antibody binding levels in saliva (final dilution 1:25) (**A** and **B**) or nasal fluid (final dilution 1:50) (**C** and **D**) against a panel of influenza HA proteins (11 influenza strains) (**A** and **C**) or SARS-CoV-2 spike proteins (XBB.1.5 and JN.1) (**B** and **D**), as measured using a bead-based multiplex assay. Vaccine strains are indicated in purple. Participants received both vaccines either in the same arm (*n* = 27; red circles) or in opposite arms (*n* = 28; blue squares). Box-and-whisker plots show the interquartile range (box), median (line), and minimum and maximum (whiskers). Experiments were performed in duplicate. Statistical significance was calculated between groups using the 2-tailed Mann-Whitney *U* test.

While influenza-specific antibodies in saliva and nasal fluid did increase following vaccination, there were no significant differences in antibody levels between the same- and opposite-arm groups across all influenza strains tested (Figure 4, A and C, and Supplemental Figures 5 and 6). As observed in blood, antibody responses to H3 Thailand 2022 following vaccination showed the largest fold-increases in saliva (5.6- vs. 3.7-fold) and nasal fluid (2.9- vs. 2.8-fold; same vs. opposite arms; Figure 4, A and C). No mucosal IgG antibodies against H5 proteins were induced (Supplemental Figure 3, B, C, E, F, H, and I).

We also observed that the fold-increase in mucosal IgG antibody responses to XBB.1.5 spike from day 0 to day 28 trended higher in the opposite-arm group in both saliva (2.5- vs. 3.7-fold) and nasal fluid (1.6- vs. 2.5-fold; same vs. opposite arms; Figure 4, B and D). However, the mucosal responses were more variable and these differences were not significant. Total IgG binding titers against XBB.1.5 and JN.1 spike proteins were not significantly different in saliva or nasal fluid at all time points tested (Supplemental Figures 5 and 6).

### Discussion

We hypothesized the ionizable lipid of the SARS-CoV-2 lipid nanoparticle mRNA vaccine could adjuvant the inactivated influenza vaccine if administered at the same site. However, our randomized controlled trial showed no difference (1.26-fold, *P* = 0.30) in HI titers to the H1, H3, and B-Vic influenza strains between the vaccine groups at day 28 after vaccination, the primary endpoint. This result was supported by multiple secondary and exploratory endpoints, including binding antibody titers to all influenza strains tested and at both day 6 and day 28 time points postvaccination.

Why did this strategy fail when it had biological plausibility from published murine studies (7)? It may be that the 2 vaccines must be coformulated in the same syringe for the lipid nanoparticle to efficiently adjuvant the influenza protein. A recent preprint suggested coformulation of influenza protein and lipid nanoparticles was superior to separate delivery within 1 hour in mice (13). Although we attempted to administer the 2 vaccines to precisely the same site in the muscle in the same arm group, we cannot be sure this was effective. Future studies coformulating the vaccines would be of interest. Another possible reason for the lack of benefit of this strategy was that we studied the recall of memory responses in a pre-immune population instead of looking at primary immunization of a naive population. The influenza vaccine in the population studied had overall modest immunogenicity (<3.17-fold median fold-increase in HI), as compared with the 256- to 1,024-fold increases in HI observed in mice with primary immunization (7). The people recruited in our study were somewhat older (median age 56) and had been given multiple prior influenza vaccinations. Younger populations with reduced exposure to influenza may respond more robustly to influenza vaccination (14). Nonetheless, older populations are a more important target population to improve influenza vaccination. The translation of animal to human studies of adjuvants is fraught with negative results owing in part to species-specific adjuvant effects. The ionizable lipid used in mouse studies is different from that in the Moderna vaccine we studied (7). Recent work illustrates combination effects of adjuvants are more helpful and may translate to humans more effectively (15). Additionally, we cannot exclude a small effect could have been missed in our 55-participant trial, though any such small effect might be of minimal clinical significance. Larger trials of less diverse populations, or giving multiple booster vaccinations, could uncover small differences in the immunogenicity of coadministration strategies. Although neutralizing antibodies are a generally accepted correlate of influenza and SARS-CoV-2 immunity, cellular immunity is also of critical importance, and exploring T and B cell immunity could generate additional hypotheses for future studies. We note our randomized trial results are consistent with recently reported observational studies (10, 11). We conclude there is no benefit to influenza immunity when influenza and SARS-CoV-2 vaccines are administered at the same site.

We did, however, observe a small detrimental effect on the fold-increase in SARS-CoV-2 antibody levels when both influenza and SARS-CoV-2 vaccines were administered at the same site. This effect was most prominent in binding antibody levels against XBB.1.5 and JN.1, but we also detected a similar detrimental effect for BA.5 and ancestral strain neutralizing antibody titers. While this finding was unexpected, we caution that this difference was relatively modest in magnitude; absolute binding and neutralizing antibody levels at day 28 postvaccination were not different between groups, and we did not correct for multiple comparisons in these secondary or exploratory endpoints. As such, it requires confirmation in other studies. Possible reasons for this effect could be (a) slight differences in baseline titers between the randomized groups or (b) a detrimental effect on the lipid nanoparticle integrity in vivo where the vaccines comingle at the same site. We note that a negative effect on SARS-CoV-2 mRNA vaccine immunogenicity was not detected in observational studies where the vaccines were presumably given at separate sites in the same arm (10, 11). Although not directly studying same- versus opposite-arm vaccination, some observational studies have reported slightly lower SARS-CoV-2 antibody titers when COVID-19 and influenza vaccines were concurrently administered compared with receiving a COVID-19 vaccine alone, possibly indicating some interference between the 2 vaccines (16). There is a possibility of antigenic competition between the 2 vaccines. Since the influenza vaccine is protein based, it may be presented earlier than the mRNA vaccine that requires translation of antigen. The potential immunological mechanisms behind changes in immunogenicity of COVID-19 vaccines when coadministered with influenza vaccines are still unknown and warrant further investigation. In vitro and animal studies examining the effect of coformulating the 2 vaccines on the integrity and/or trafficking of the SARS-CoV-2 vaccine would also be of interest.

In conclusion, our randomized trial administering an influenza vaccine at the same site as a SARS-CoV-2 mRNA lipid nanoparticle vaccine did not improve influenza immunity and may have led to a small detrimental effect on SARS-CoV-2 immunity. Further studies are warranted but in the interim, when the 2 vaccines are administered on the same day, it may be preferable to give them in separate arms or at least some distance apart if given in the same arm.

## Methods

### Sex as a biological variable.

Male and female participants were enrolled in this study, which was open to all sexes. Randomization included matching for sex.

### Study design.

The CANNON study was an open-label, randomized trial of administering both an influenza and COVID-19 vaccine on the same day in the same arm or in opposite arms. Healthy adults (18–65 years) who had received 2 or more prior doses of COVID-19 vaccines at least 4 months before recruitment were eligible. Exclusion criteria included prior COVID-19 infection within 4 months, immunosuppression, previous significant adverse events to influenza or COVID-19 vaccines, and prior anaphylaxis of any cause. Participants were recruited in Melbourne, Australia, and were randomized to receive the 2024 CSL Seqirus quadrivalent unadjuvanted influenza vaccine (Afluria) and Moderna Omicron XBB.1.5–containing COVID-19 mRNA booster vaccine (Spikevax) administered intramuscularly in either the same arm or opposite arms. The same-arm group received both vaccinations in the nondominant arm at the same injection site using separate needles and syringes. The opposite-arm group received the Moderna COVID-19 mRNA vaccine in the nondominant arm and the Afluria influenza vaccine in the dominant arm. Blood and saliva samples (SalivaBio; Salimetrics) were collected prior to vaccination and days 6 and 28 postvaccination. Nasal fluid samples were collected prior to vaccination and day 28 postvaccination (Nasorption FX-i; Mucosal Diagnostics) (17). Adverse event data were collected on day 6.

The primary endpoint was the difference in postvaccination HI titer combined to 3 influenza vaccine strains (H1N1, H3N2, and B-Vic) at day 28 in the same-arm group compared with the opposite-arm group. Based on HI titer data from 114 participants receiving vaccination in 2023, we estimated the postvaccination HI titer standard deviation is 0.37 on a log_10_ scale. Calculating a geometric mean across the 3 influenza strains, we estimated we had 80% statistical power to detect a 2-fold change (a 0.3 log_10_ difference) in the same-arm group compared with the opposite-arm group (with statistical significance of 0.05), using a minimum of 25 participants in each group. We considered a 2-fold change in HI likely to be a meaningful increase in protective immunity. Dynamic (adaptive) randomization with minimization to promote balance in age, sex, and 2023 influenza immunization was used to allocate participants to either interventional group. Age was stratified by 10-year intervals. This was achieved using R, library Minirand, function Minirand, using equal weighting of covariate factors and high probability of assignment = 0.90. Key additional endpoints included influenza antibody responses in plasma, saliva, and nasal fluids; antibody responses to other influenza strains; SARS-CoV-2 antibody responses; and self-reported adverse events collected at day 6.

### Immunologic assays.

An HI assay was conducted at the WHO Collaborating Centre for Reference and Research on Influenza as previously described (18) using the 4 vaccine influenza strains as listed in Supplemental Table 1. A bead-based multiplex array containing both influenza HA and SARS-CoV-2 spike proteins (as listed in Supplemental Table 1) was conducted, as previously described (12), to measure antibody responses in plasma, saliva, and nasal fluid (details and validation in Supplemental Figure 1). A live virus SARS-CoV-2 neutralization assay was conducted as previously described (19) using strains listed in Supplemental Table 1. Assays were performed in duplicate.

### Statistics.

Antibody responses postvaccination to influenza and SARS-CoV-2 at day 28 and the fold-change in the responses from day 0 to day 28 were compared between the same- and opposite-arm group. When titers were averaged across variants or individuals, geometric means were used. For SARS-CoV-2 neutralization titers, many values were below the limit of detection; this was accounted for using a censored regression analysis (performed in R v4.3.1 using the censReg functions). In all box-and-whisker plots, the central horizontal line indicates the median value, the box indicates the interquartile range, and the whiskers indicate the range of the data. Comparisons of antibody titers, fold-changes, or mean titers (across variants) between groups were analyzed using 2-tailed Mann-Whitney *U* tests with the wilcox.test function in R (v4.3.1). Spearman’s correlations were used to assess the relationship of IgG antibody responses across plasma, saliva, and nasal fluid samples with the cor.test function in R. The Fisher exact test was used to compare the proportion of participants with local reaction in the same- and opposite-arm groups. *P* values of 0.05 or less were considered significant. All reported *P* values are raw *P* values without adjusting for multiple comparisons.

### Study approval.

This study was approved by the University of Melbourne Human Research Ethics Committee (approval no. 28318). Written informed consent was obtained from all participants prior to enrollment in the study. This study was registered with the Australian New Zealand Clinical Trials Registry (ID ACTRN12624000445572).

### Data availability.

All the data and methods are presented in the manuscript or in the supplemental materials. All individual values for figures are available in the Supporting Data Values file.

## Author contributions

SJK conceived and designed the study. HEK and SJK recruited the study participants. TES generated the random allocation sequence and assigned participants to the interventions. WSL, KJS, JA, HP, MA, and IB were responsible for the acquisition of data. KJS, WSL, JA, AR, MPD, and SJK performed the analyses and interpreted the results. SJK, KJS, WSL, AR, and JA wrote the first draft of the manuscript. WSL, KJS, JA, HEK, AR, TES, DC, DSK, HP, MA, MNV, MZMZ, AWC, MK, HXT, AKW, JAJ, SR, MPD, IB, and SJK contributed intellectually to the work, critically revised the report, and approved the final version. The order of co–first authors’ names was assigned on the basis of their experimental and editorial contributions to this study.

## Supplementary Material

Supplemental data

ICMJE disclosure forms

Supporting data values

## Figures and Tables

**Table 1:**
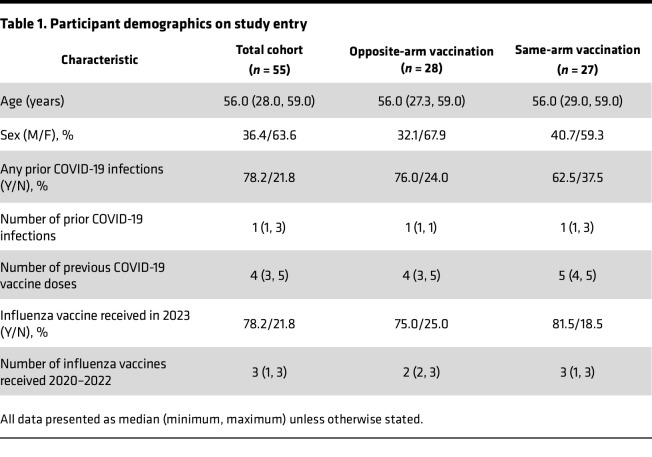
Participant demographics on study entry

**Table 2:**
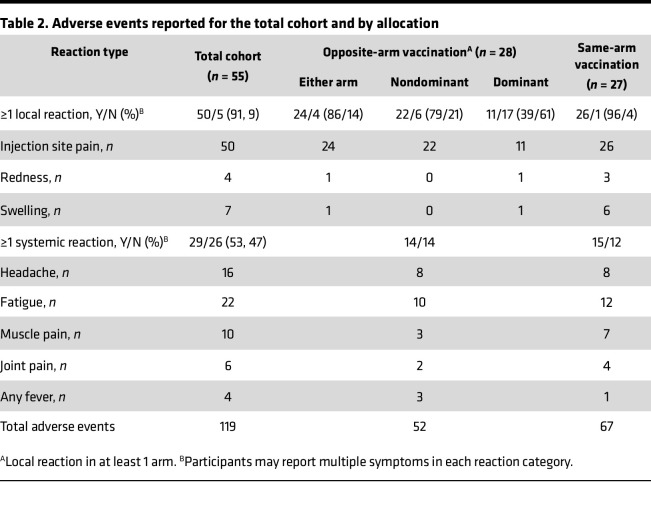
Adverse events reported for the total cohort and by allocation

## Version 1 01/09/2025

In-Press Preview

## Version 2 02/24/2025

Electronic publication
