# Supplementary material for: Randomized trial of same- versus opposite-arm coadministration of inactivated influenza and SARS-CoV-2 mRNA vaccines
Source: JCI Insight. 2025 Jan 9;10(4):e187075. doi: 10.1172/jci.insight.187075 (PMC11949032; doi:10.1172/jci.insight.187075)
Supplement: Supplemental data [file jciinsight-10-187075-s053.pdf]

Supplementary information

Randomized trial of same vs opposite arm co-administration of inactivated influenza and SARS-CoV-2 mRNA vaccines

Wen Shi Lee, Kevin J. Selva, Jennifer Audsley, Helen E. Kent, Arnold Reynaldi, Timothy E. Schlub, Deborah Cromer, David S. Khoury, Heidi Peck, Malet Aban, Mai Ngoc Vu, Ming Z. M. Zheng, Amy W. Chung, Marios Koutsakos, Hyon Xhi Tan, Adam K. Wheatley, Jennifer A. Juno, Steven Rockman, Miles P. Davenport, Ian Barr, Stephen J. Kent

Supplemental Figure 1

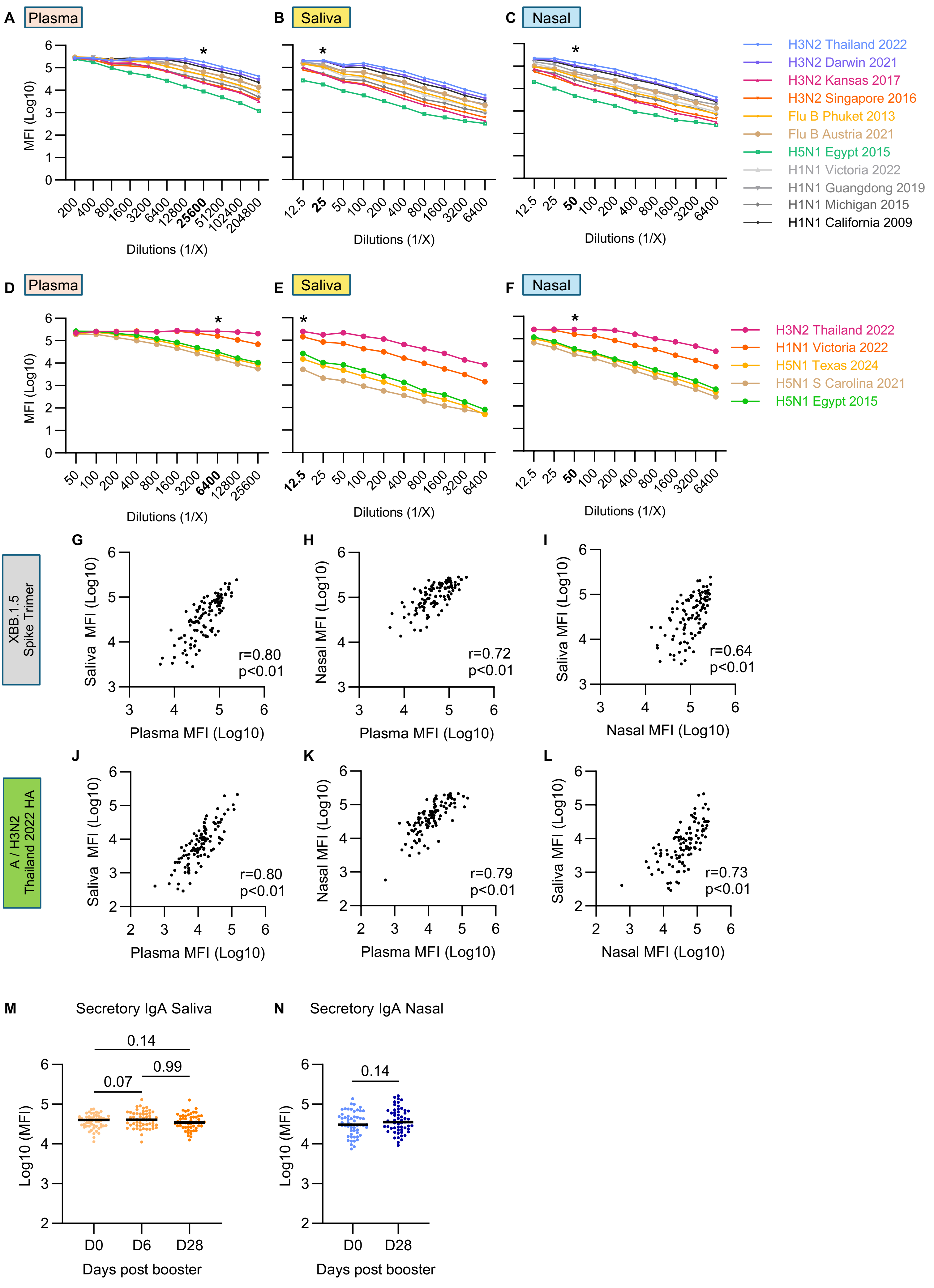

Supplemental Figure 1: Multiplex assay titrations and control assays. (A-C) Line graphs illustrate the titration curves for the main influenza HA multiplex array with plasma **(A)**, saliva **(B)**, and nasal fluid **(C)**. **(D-F)** Line graphs also show the titration curves for plasma **(D)**, saliva **(E)**, and nasal fluid **(F)** with the influenza HA sub-analysis array using more concentrated dilutions for increased sensitivity to detect H5N1 responses. Working dilutions chosen for each sample type and array are shown with asterisks and bold text. Scatter plots display the Spearman correlations of IgG antibodies in plasma, saliva and nasal samples binding to XBB.1.5 Spike **(G-I)** and influenza A H3N2 Thailand 2022 HA **(J-L)** respectively across the entire cohort. The quality of both saliva **(M)** and nasal fluid **(N)** samples collected throughout the study were also shown to be consistent as depicted by the tight clustering of total secretory IgA measured across collection timepoints. Experiments were performed in duplicate. Statistical significance was calculated between groups using the 2-tailed Friedman and Wilcoxon tests respectively. *P* values of >0.05 were considered non-significant

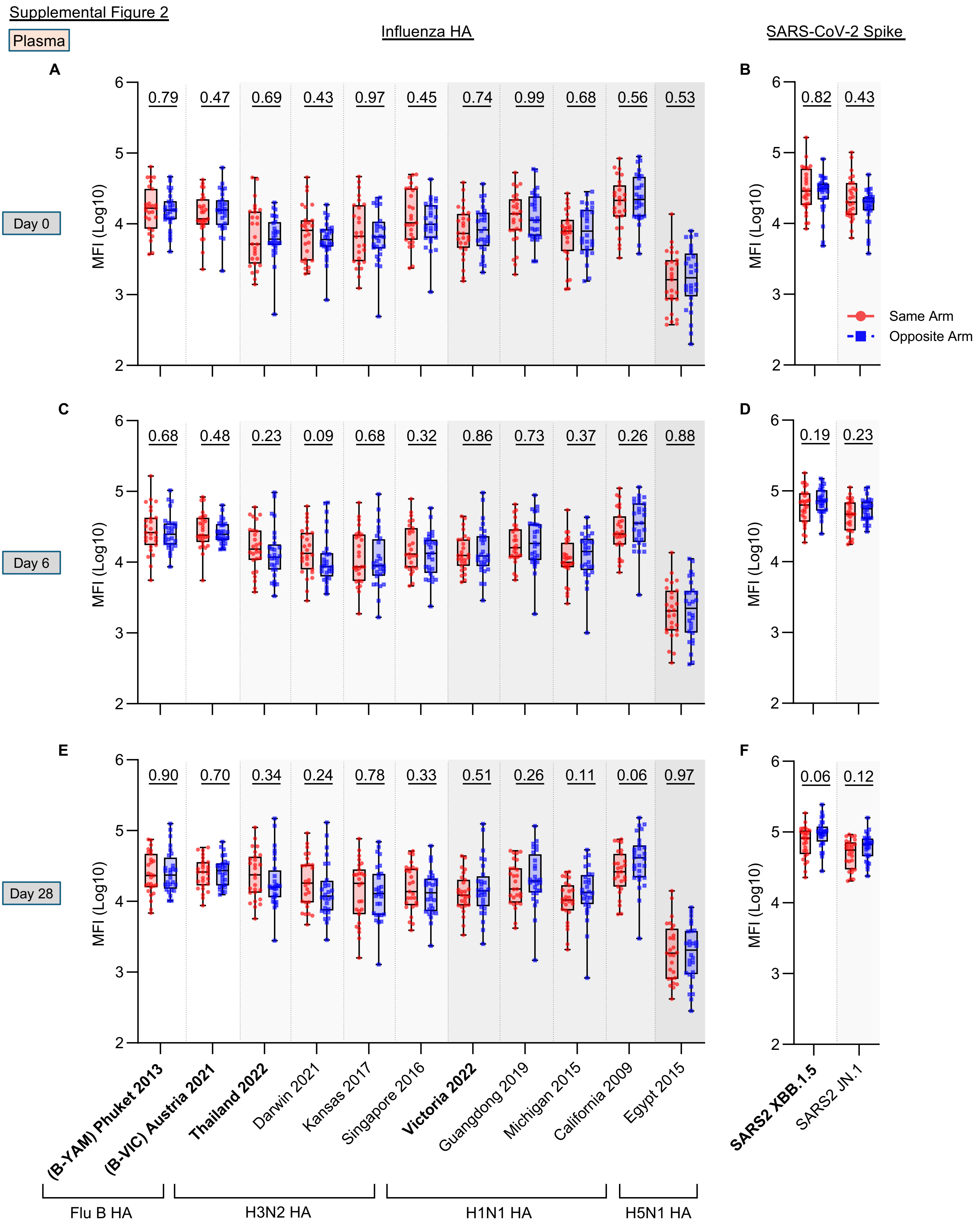
Supplemental Figure 2
Plasma
Influenza HA
SARS-CoV-2 Spike
A
B
C
D
E
F
Day 0
Day 6
Day 28
MFI (Log10)
0.79
0.47
0.69
0.43
0.97
0.45
0.74
0.99
0.68
0.56
0.53
0.82
0.43
0.68
0.48
0.23
0.09
0.68
0.32
0.86
0.73
0.37
0.26
0.88
0.19
0.23
0.90
0.70
0.34
0.24
0.78
0.33
0.51
0.26
0.11
0.06
0.97
0.06
0.12
Same Arm
Opposite Arm
(B-YAM) Phuket 2013
(B-VIC) Austria 2021
Thailand 2022
Darwin 2021
Kansas 2017
Singapore 2016
Victoria 2022
Guangdong 2019
Michigan 2015
California 2009
Egypt 2015
SARS2 XBB.1.5
SARS2 JN.1
Flu B HA
H3N2 HA
H1N1 HA
H5N1 HA

Supplemental Figure 2: Plasma IgG antibody binding responses to influenza HA and SARS-CoV-2 spike following vaccination. Plasma IgG antibody binding to either influenza HA (11 different influenza strains) (**A, C and E**) or SARS-CoV-2 spike proteins (XBB.1.5 and JN.1) **(B, D and F)** at days 0 **(A and B)**, 6 **(C and D)** and 28 **(E and F)** post-vaccination respectively were measured using a bead-based multiplex assay (final dilution 1:25600). Vaccine strains are indicated in bold. Subjects received both vaccines in either the same (n=27; red circles) or opposite arms (n=28; blue squares). Box plots show the interquartile range (box), median (line), and minimum and maximum (whiskers). Experiments were performed in duplicate. Statistical significance was calculated between groups using the 2-tailed Mann-Whitney *U* test. *P* values of >0.05 were considered non-significant.

Supplemental Figure 3

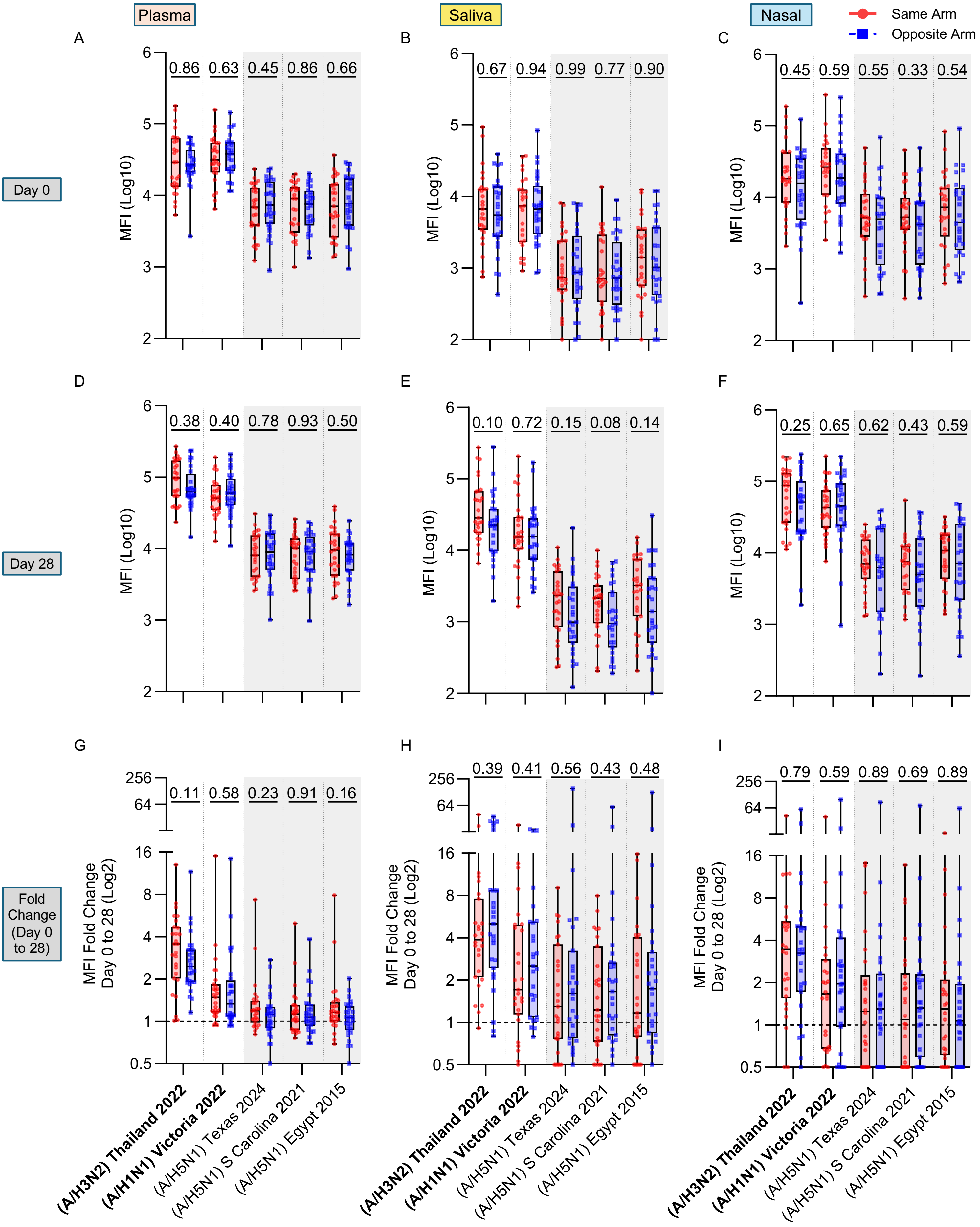

Supplemental Figure 3: Antibody binding responses in plasma, saliva and nasal fluid to circulating H5N1 influenza strains. IgG levels in plasma **(A, D)** (final dilution 1:6400), saliva **(B, E)** (final dilution 1:12.5) and nasal fluid **(C, F)** (final dilution 1:25) against influenza HA proteins from 5 different influenza strains were measured using a bead-based multiplex assay at days 0 **(A-C)** and 28 **(D-F)** post-vaccination. Vaccine strains are indicated in bold. Subjects received both vaccines in either the same (n=27; red circles) or opposite arms (n=28; blue squares). Box plots show the interquartile range (box), median (line), and minimum and maximum (whiskers). Experiments were performed in duplicate. Statistical significance was calculated between groups using the 2-tailed Mann-Whitney *U* test. *P* values of >0.05 were considered non-significant.

Supplemental Figure 4

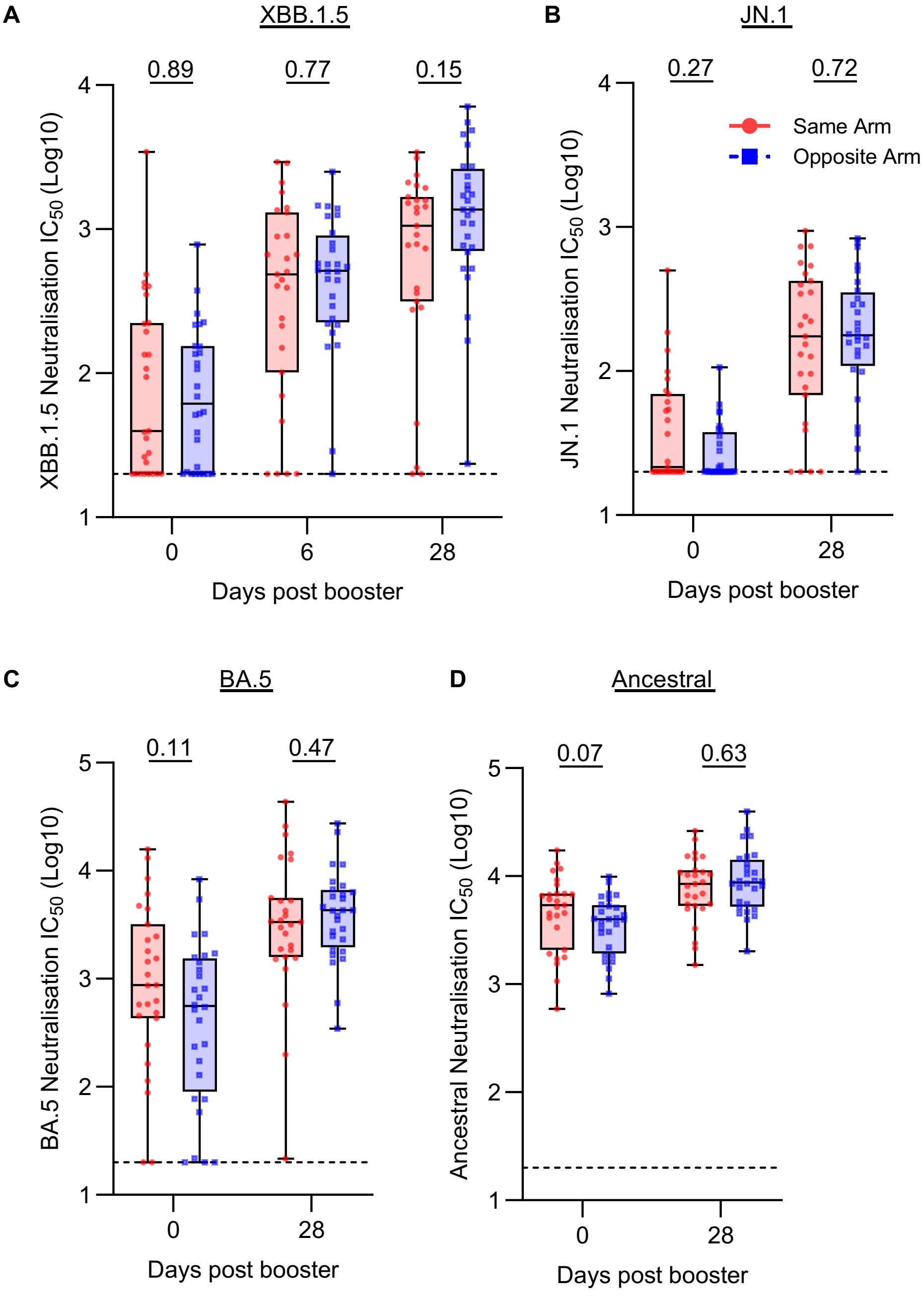

Supplemental Figure 4: Plasma neutralising antibody titres against SARS-CoV-2 strains. Box and whiskers plots depict plasma neutralising antibody titres against **(A)** XBB.1.5, **(B)** JN.1, **(C)** BA.5 and **(D)** ancestral SARS-CoV-2 strains for up to 28 days post vaccination. Subjects either received both vaccines in the same (n=27; red circles) or opposite arms (n=28; blue squares). Box plots show the interquartile range (box), median (line), and minimum and maximum (whiskers). Experiments were performed in duplicate. Statistical significance was calculated between groups using the 2-tailed Mann-Whitney *U* test. *P* values of >0.05 were considered non-significant.

Supplemental Figure 5

Saliva

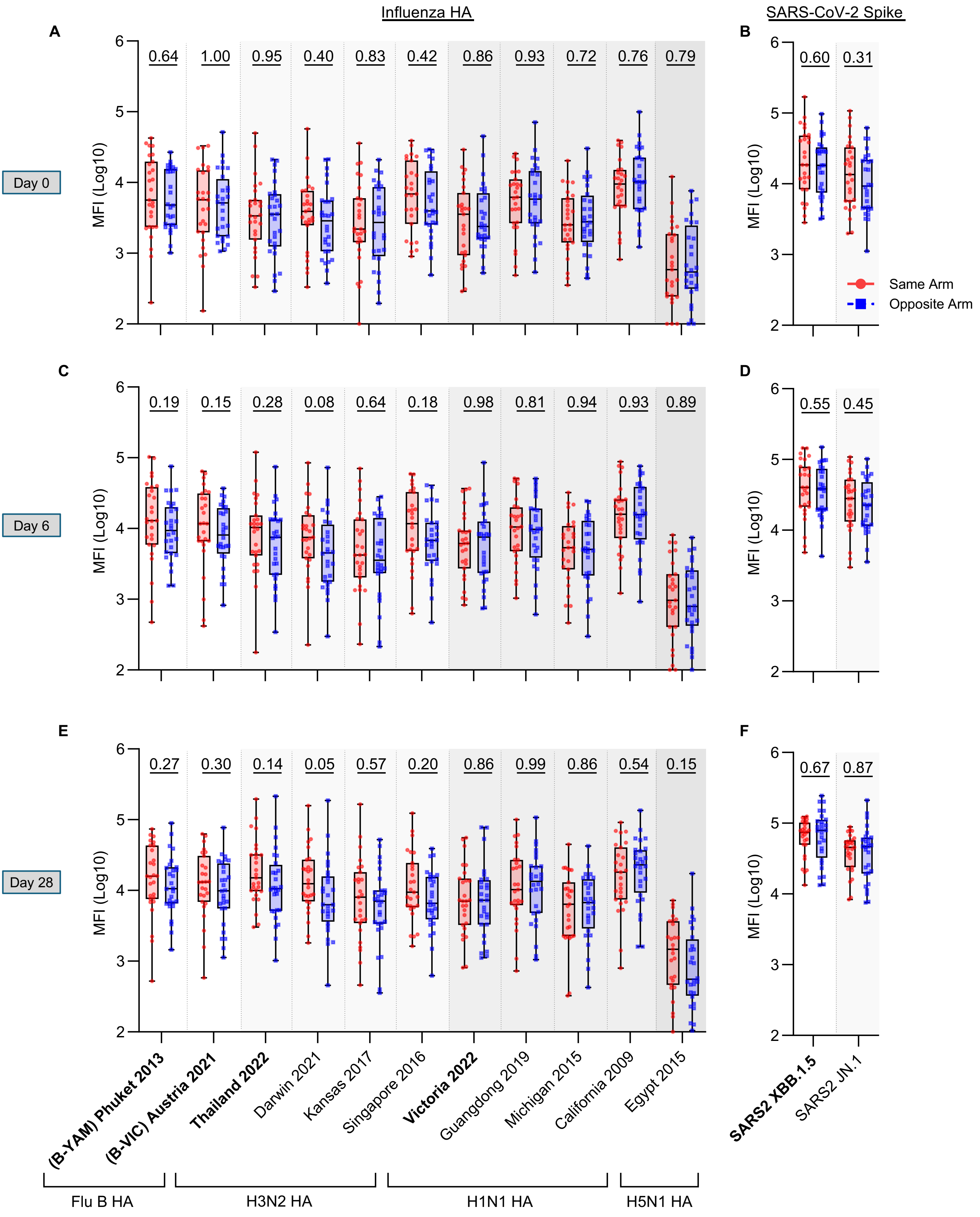

Supplemental Figure 5: Salivary IgG antibody binding responses to influenza HA and SARS-CoV-2 spike following vaccination. Salivary IgG antibody binding to either influenza HA (11 different influenza strains) (**A, C and E**) or SARS-CoV-2 spike proteins (XBB.1.5 and JN.1) **(B, D and F)** at days 0 **(A and B)**, 6 **(C and D)** and 28 **(E and F)** post-vaccination respectively were measured using a bead-based multiplex assay (final dilution 1:25). Vaccine strains are indicated in bold. Subjects received both vaccines in either the same (n=27; red circles) or opposite arms (n=28; blue squares). Box plots show the interquartile range (box), median (line), and minimum and maximum (whiskers). Experiments were performed in duplicate. Statistical significance was calculated between groups using the 2-tailed Mann-Whitney *U* test. *P* values of >0.05 were considered non-significant.

Supplemental Figure 6

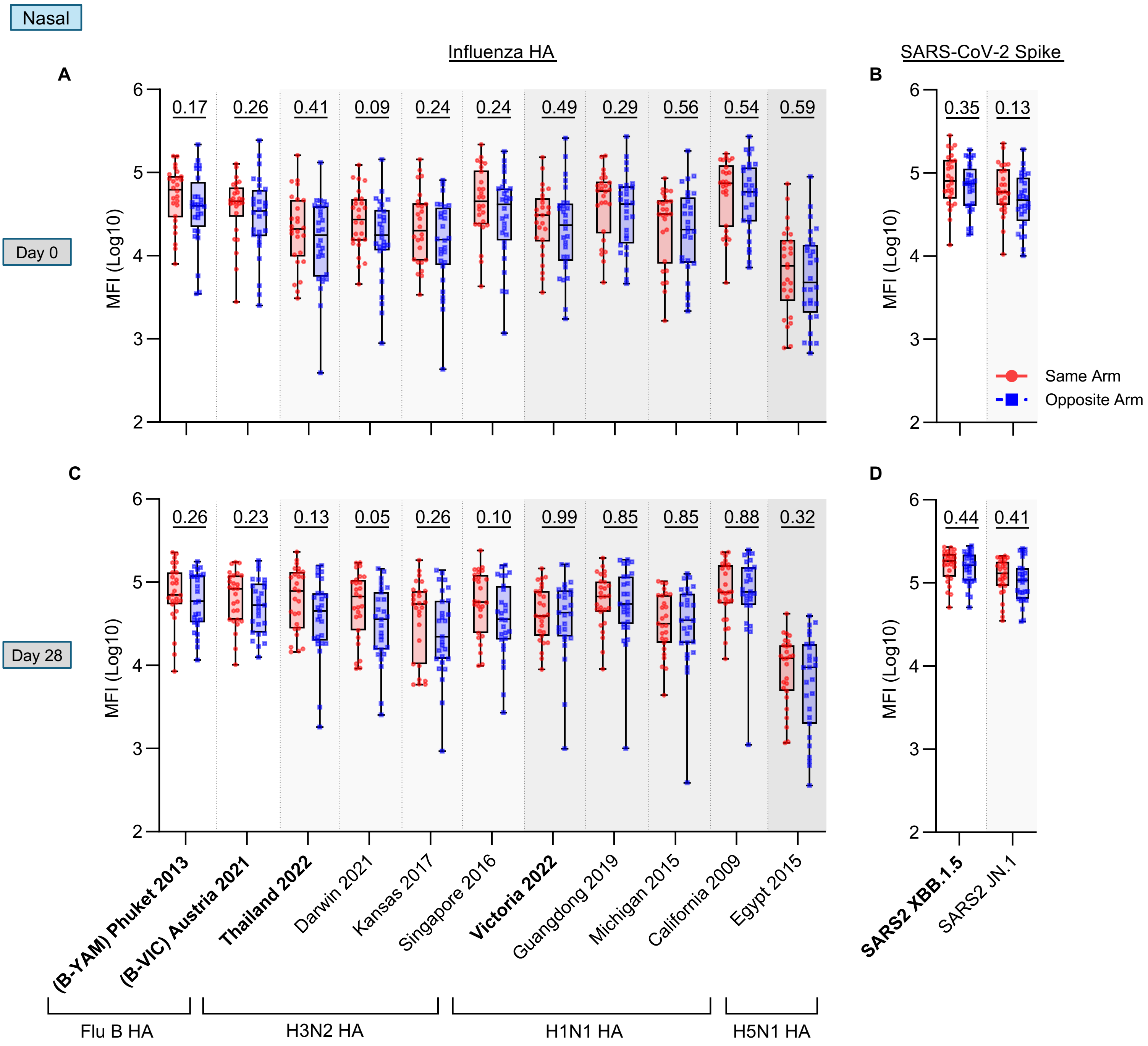

Supplemental Figure 6: IgG antibody binding responses to influenza HA and SARS-CoV-2 spike in nasal fluid following vaccination. Nasal IgG antibody binding to either influenza HA (11 different influenza strains) (**A and C**) or SARS-CoV-2 spike proteins (XBB.1.5 and JN.1) **(B and D)** at days 0 **(A and B)** and 28 **(C and D)** post-vaccination respectively were measured using a bead-based multiplex assay (final dilution 1:50). Vaccine strains are indicated in bold. Subjects received both vaccines in either the same (n=27; red circles) or opposite arms (n=28; blue squares). Box plots show the interquartile range (box), median (line), and minimum and maximum (whiskers). Experiments were performed in duplicate. Statistical significance was calculated between groups using the 2-tailed Mann-Whitney *U* test. *P* values of >0.05 were considered non-significant.

Supplemental Table 1 – Virus reference strains and multiplex array proteins

**Influenza strains**			
IVR-238 (A/Victoria/4897/2022)			
BVR-26 (B/Austria/1359417/2021)			
BVR-1B (B/Phuket/3073/2013)			
IVR-237 (A/Thailand/8/2022)			
**SARS-CoV-2 strains**	**Identifier**		
Ancestral D614G	hCoV-19/Australia/VIC31/2020		
Omicron BA.5	hCoV-19/Australia/VIC61194/2022		
Omicron XBB.1.5	hCoV-19/Australia/VIC65076/2023		
Omicron JN.1	hCoV-19/Australia/VIC-VIDRL-00012759/2023		
**Recombinant proteins**	**Cat #**	**Expressed Host**	**Company / Source**
Influenza A/Thailand/8/2022 (H3N2) HA	40992-V08H	HEK293	Sino Biological
Influenza A/Darwin/9/2021 (H3N2) HA	40859-V08H	HEK293	Sino Biological
Influenza A/Kansas/14/2017 (H3N2) HA	40720-V08H	HEK293	Sino Biological
Influenza A/Singapore/infimh/16-0019/2016 (H3N2) HA	40580-V08H	HEK293	Sino Biological
Influenza A/Victoria/4897/2022 (H1N1) HA	40938-V08H	HEK293	Sino Biological
Influenza A/Guangdong-Maonan/SWL1536/2019 (H1N1) HA	40717-V08H	HEK293	Sino Biological
Influenza A/Michigan/45/2015 (H1N1) HA	40567-V08H1	HEK293	Sino Biological
Influenza A/California/7/2009 (H1N1) HA	11085-V08H	HEK293	Sino Biological
Influenza A/Texas/37/2024 (H5N1) HA	41036-V08H	HEK293	Sino Biological
Influenza A/American wigeon/South Carolina/AH0195145/2021 (H5N1) HA	41013-V08H1	HEK293	Sino Biological
Influenza A/Egypt/N0001/2015 (H5N1) HA	40699-V08H	HEK293	Sino Biological
Influenza B/Austria/1359417/2021 (VIC) HA	40862-V08H	HEK293	Sino Biological
Influenza B/PHUKET/3073/2013 (YAM) HA	HAE-V52H4-100ug	HEK293	Acro Biosystems
SARS-CoV-2 XBB.1.5 Spike Trimer	40589-V08H45	HEK293	Sino Biological
SARS-CoV-2 JN.1 Spike Trimer	40592-V08H155	HEK293	Sino Biological
SIV gp120 (negative control)	40415-V08H	HEK293	Sino Biological

HA: Hemagglutinin
